# Mechanistic insights into spontaneous transition from cellular alternans to ventricular fibrillation

**DOI:** 10.14814/phy2.15619

**Published:** 2023-03-02

**Authors:** Tingting You, Yulong Xie, Cunjin Luo, Kevin Zhang, Henggui Zhang

**Affiliations:** ^1^ Key Laboratory of Medical Electrophysiology, Ministry of Education and Medical Electrophysiological Key Laboratory of Sichuan Province, (Collaborative Innovation Center for Prevention of Cardiovascular Diseases) Institute of Cardiovascular Research, Southwest Medical University Luzhou China; ^2^ Department of Neurosurgery Xinqiao Hospital, Army Medical University Chongqing China; ^3^ School of Computer Science and Electronic Engineering University of Essex Colchester UK; ^4^ School of Medicine Imperial College of London London UK; ^5^ Department of Physics and Astronomy University of Manchester Manchester UK

**Keywords:** arrhythmia, impaired repolarization, optical mapping, patch clamp, spatially discordant alternans, sudden cardiac death, T‐wave alternans

## Abstract

T‐wave alternans (TWA) has been used for predicting the risk of malignant cardiac arrhythmias and sudden cardiac death (SCD) in multiple clinical settings; however, possible mechanism(s) underlying the spontaneous transition from cellular alternans reflected by TWA to arrhythmias in impaired repolarization remains unclear. The healthy guinea pig ventricular myocytes under E‐4031 blocking *I*_Kr_ (0.1 μM, *N* = 12; 0.3 μM, *N* = 10; 1 μM, *N* = 10) were evaluated using whole‐cell patch‐clamp. The electrophysiological properties of isolated perfused guinea pig hearts under E‐4031 (0.1 μM, *N* = 5; 0.3 μM, *N* = 5; 1 μM, *N* = 5) were evaluated using dual‐ optical mapping. The amplitude/threshold/restitution curves of action potential duration (APD) alternans and potential mechanism(s) underlying the spontaneous transition of cellular alternans to ventricular fibrillation (VF) were examined. There were longer APD_80_ and increased amplitude and threshold of APD alternans in E‐4031 group compared with baseline group, which was reflected by more pronounced arrhythmogenesis at the tissue level, and were associated with steep restitution curves of the APD and the conduction velocity (CV). Conduction of AP alternans augmented tissue's functional spatiotemporal heterogeneity of regional AP/Ca alternans, as well as the AP/Ca dispersion, leading to localized uni‐directional conduction block that spontaneous facilitated the formation of reentrant excitation waves without the need for additional premature stimulus. Our results provide a possible mechanism for the spontaneous transition from cardiac electrical alternans in cellular action potentials and intercellular conduction without the involvement of premature excitations, and explain the increased susceptibility to ventricular arrhythmias in impaired repolarization. In this study, we implemented voltage‐clamp and dual‐optical mapping approaches to investigate the underlying mechanism(s) for the arrhythmogenesis of cardiac alternans in the guinea pig heart at cellular and tissue levels. Our results demonstrated a spontaneous development of reentry from cellular alternans, arising from a combined actions of restitution properties of action potential duration, conduction velocity of excitation wave and interplay between alternants of action potential and the intracellular Ca handling. We believe this study provides new insights into underlying the mechanism, by which cellular cardiac alternans spontaneously evolves into cardiac arrhythmias.

## Graphical abstract

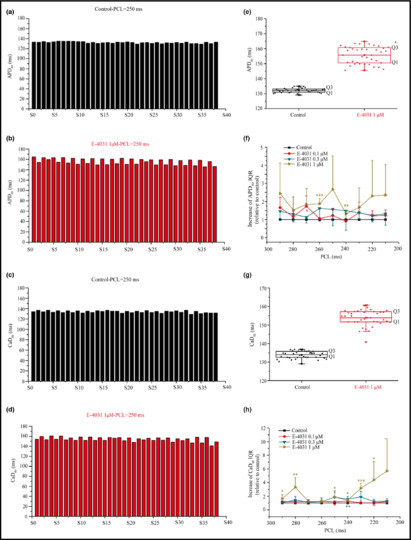

## 1 INTRODUCTION

Previous clinical studies have revealed that T‐wave alternans (TWA) is related to the pathophysiological mechanism and clinical prognosis of sudden cardiac death (SCD; Pang et al., 2019; Zipes et al., 2006). It is associated with increased risk of cardiac arrhythmogenesis in many heart disease conditions, such as long QT syndromes (LQTS; Akar et al., 2002; Huang et al., 2016; Liu & Laurita, 2005), acute myocardial infarction (Huang et al., 2018), heart failure (Fukaya et al., 2019), and catecholaminergic polymorphic ventricular tachycardia (Yang et al., 2016). However, the mechanism of the spontaneous transition from TWA to arrhythmias remains unknown.

At the cellular level, TWA can be attributable to alternans of action potential morphology (AP alternans) and/or cytosolic calcium transient (CaT alternans). The possible hypotheses underlying AP/CaT alternans have been investigated by both simulation and experimental studies (Huang et al., 2020; Osadchii, 2019; Wang et al., 2018). One of the most well‐known hypotheses is the action potential duration (APD) restitution theory (Nolasco & Dahlen, 1968; Shattock et al., 2017), attributing the genesis and maintenance of APD alternans to the maximum slope of the APD restitution curve. When the maximum slope of the APD restitution curve is greater than one, stable APD alternans and complex APD alterations may occur (Nolasco & Dahlen, 1968). However, owing to the effect of cardiac excitatory memory, some other studies (Comlekoglu & Weinberg, 2017; Goldhaber et al., 2005) have revealed that the APD restitution theory alone is insufficient to explain stable APD alternans, which may involve more complex dynamical processes. Another hypothesis is the CaT alternans, which mainly involves the unstable release and sequestration of calcium in the sarcoplasmic reticulum (SR; Weinberg, 2016) or mitochondrial dysfunctions (Oropeza‐Almazan & Blatter, 2020). Through mechano‐electrical coupling, CaT alternans can be manifested as APD alternans, leading to TWA on the electrocardiogram (ECG). Due to the bi‐directional coupling between membrane voltage and calcium handling dynamics, it is impossible to explore which is the main or the secondary determinant between the two.

At the tissue level, cellular AP alternans can be manifested as spatially concordant and/or discordant conduction alternans (SCA/SDA; Colman, 2020; Munoz et al., 2018). By its nature, SDA has the potential to produce larger spatial repolarization dispersion than SCA, thus promoting uni‐directional conduction block and leading to reentry. As of now, possible mechanisms underlying the pro‐arrhythmogenesis of SCA and/or SDA may result from the amplification of pre‐existing tissue heterogeneity by the SCA/SDA‐induced functional heterogeneity of cardiac tissue leading to substrates favoring the initiation and maintenance of arrhythmias (Pastore et al., 2006). However, some experimental and simulation studies showed that pre‐existing tissue heterogeneities may not be necessary for the formation of SDA predicting arrhythmogenesis (Huang et al., 2020; Watanabe et al., 2001). Substantial studies suggested that dynamic properties, such as the steep APD restitution (Nolasco & Dahlen, 1968) and conduction velocity (CV) restitution (Huang et al., 2020; Wang et al., 2018), intracellular calcium cycling instability (Song et al., 2018; Sun et al., 2018), and autonomic nervous system regulation (Winter et al., 2018; Xiong et al., 2018) can convert SCA into SDA to facilitate arrhythmogenesis. Up to date progress on the experimental approaches to investigate potential mechanism(s) underlying TWA at the cellular and tissue level has been extensively reviewed (Wilson & Rosenbaum, 2007; You et al., 2021). Though previous experimental studies (Huang et al., 2016, 2020; Lau et al., 2015; Liu et al., 2018; Munoz et al., 2018; Nemec et al., 2016; Sato et al., 2006; Song et al., 2018; Sun et al., 2018; Tse et al., 2016; Watanabe et al., 2001; Winter et al., 2018; Xiong et al., 2018) have suggested that the augmented electrical heterogeneity in tissue by SDA forms a pro‐arrhythmic substrate facilitating the formation of reentrant excitation waves, extra premature stimuli (mimicking ectopic foci, DAD or EADs) were applied to initiate reentry in those studies. To our best knowledge, potential mechanism(s) underlying the spontaneous transition of cellular alternans to ventricular fibrillation (VF) without the involvement of premature excitations and particularly in impaired repolarization remains to be unclear. Our previous simulation study (Wang et al., 2018) predicted that in a *I*_Na_‐defected tissue a spontaneous transition from cellular electrical alternans to reentrant excitation at the tissue level is possible, but experimental evidence to support this prediction is missing.

In this study, we used whole‐cell patch clamp and dual‐optical mapping experiments to investigate experimentally the mechanisms underlying the spontaneous transition from cellular AP/CaT alternans to reentrant arrhythmia under normal and impaired repolarization condition mimicked by administration of E‐4031 that blocks *I*_Kr_ in the adult guinea pig ventricle. This study, an acute impaired repolarization model mimiced by administrating E‐4031, manifested as prolonged APD and QT interval in the ECG. In impaired repolarization, AP alternans was more susceptible to be induced in ventricular myocytes and isolated guinea pig hearts. In addition, cardiac alternans is more stable and easier to transit from SCA to SDA in impaired repolarization due to the steeper APD restitution and the CV restitution curves as compared to the control condition. Such conduction alternans increased tissue's spatiotemporal heterogeneity of regional APD/CaT duration (CaD), leading to augmented functional APD/CaD dispersion that increased susceptibility of uni‐directional conduction failure or block, forming a substrate conducive to the occurrence of arrhythmia in impaired repolarization. This study adds first hands experimental evidence in showing how spontaneous transition from cellular alternans to VF can occur without the involvement of premature stimulus, especially in the condition with the administration of E‐4031, which helps to explain the increased susceptibility to ventricular arrhythmias in impaired repolarization without the need of EAD/DAD or ectopic focal activity as a trigger.

## 2 MATERIALS AND METHODS

### 2.1 Experimental animals and preparations

All experiments were performed in accordance with the Guide for the Care and Use of Laboratory Animals, National Institutes of Health, and approved by the Animal Care and Use Committee at the Southwest Medical University.

### 2.2 Myocyte isolation

Adult male guinea pigs (240–340 g) were obtained from Southwest Medical University. The animals were housed (4 per cage) under conditions of controlled humidity (55%–65%) and temperature (23–25°C) with a 12‐h dark/light cycle. Guinea pig hearts were isolated by thoracotomy after intraperitoneal injection of heparin (3125 UI/kg) and sodium pentobarbital (50 mg/kg). The depth of the anesthesia was evaluated by foot pinch to assure a deep anesthesia and that the animal does not experience any pain. In single cell experiments, hearts were excised rapidly, and ventricular myocytes were obtained by Langendorff enzymatic digestion. Excised hearts were mounted on a Langendorff apparatus (Harvard Apparatus) and retrogradely perfused via the aorta. After an initial 2–3 min perfusion with oxygenated (100% O_2_) Tyrode solution containing (mmol/L): 140 NaCl, 1 MgCl_2_, 5 KCl, 5 HEPES, 10 D‐Glucose, 1.8 CaCl_2_ (pH adjust to 7.35 with NaOH) in constant flow rate (8 mL/min), Ca^2+^‐free Tyrode solution was used to perfuse the heart for 8–10 min, followed by a digestive solution containing 0.02% collagenase (Type II, Worthington Biochemical) and 0.1% BSA. When the heart became softened, the whole ventricle was dissected and minced in an oxygenated (100% O_2_) KB (high‐K^+^) solution containing (mmol/L): 120 K‐glutamate, 20 D‐Glucose, 10 KCl, 10 KH_2_PO_4_, 10 taurine, 10 HEPES, 10 mannitol, 1.8 MgSO_4_, 0.5 EGTA, as well as 0.2% BSA (pH adjust to 7.3 with KOH) at room temperature.

### 2.3 Isolated whole heart preparation

Guinea pig hearts were rapidly excised, mounted on a Langendorff apparatus, and retrogradely perfused via the aorta. The heart was perfused with modified Krebs–Henseleit (KH) solution containing (mmol/L): 119 NaCl, 25 NaHCO_3_, 10 D‐Glucose, 4 KCl, 1.8 CaCl_2_, 1.2 KH_2_PO_4_, and 1 MgCl_2_. The solution was continuously oxygenated with a mixed gas (95% O_2_/5% CO_2_) with the flow rate of 8 mL/min at 37 ± 0.2°C. Isolated hearts were perfused and monitored for stability about 20 mins before the following experiment.

### 2.4 Patch clamp

**FIGURE 1:**
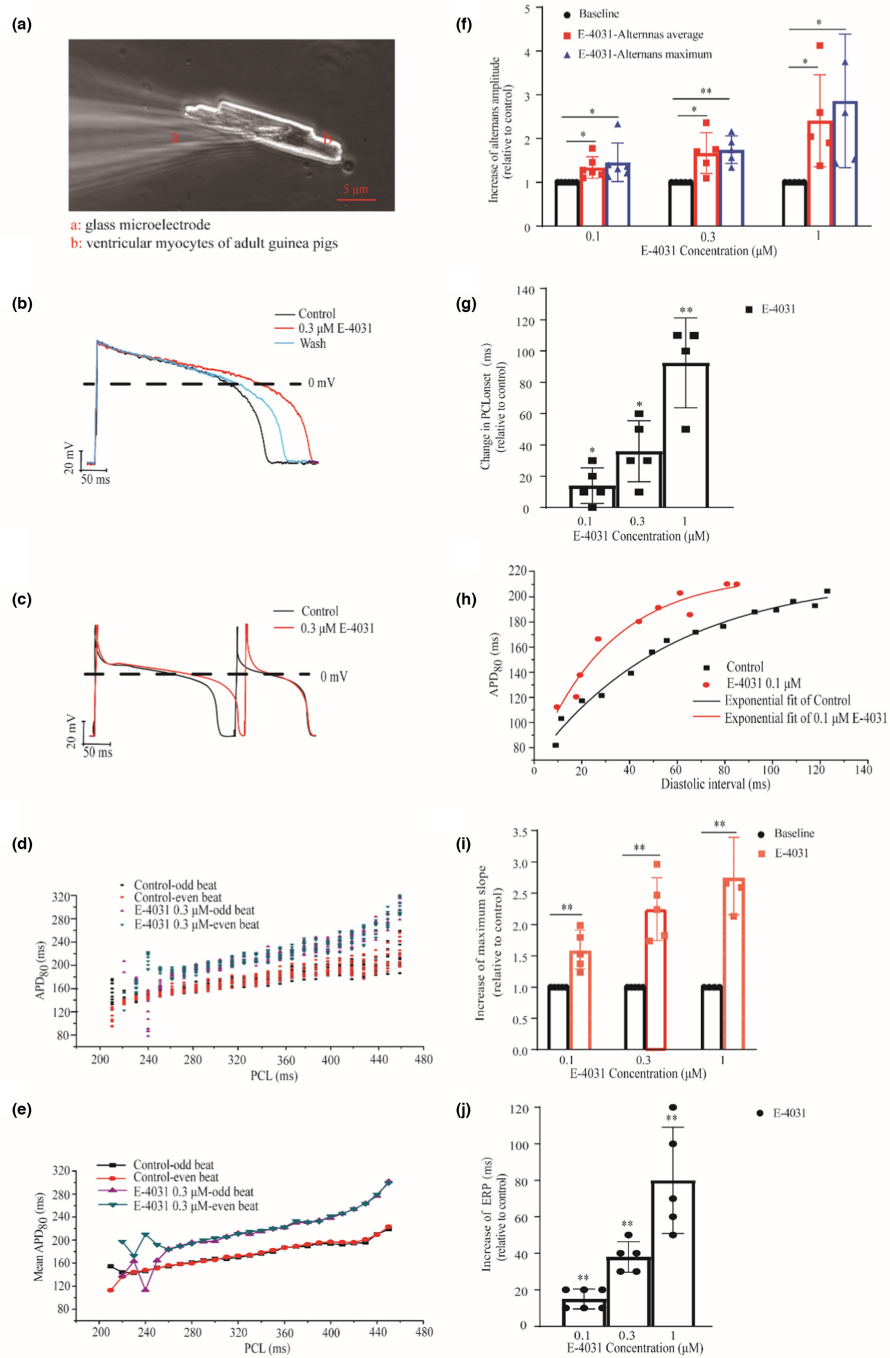
AP alternans generated in control and impaired repolarization condition. As compared to control, AP alternans is more susceptible to be induced at the cellular level. (a) Representative ventricular myocytes of adult guinea pigs (after re‐introducing Ca^2+^ to 1.8 mM, 20×) for whole‐cell patch clamp recording. (b) Representative AP recordings (1 Hz) in control, 0.3 μM E‐4031 and washout. (c) Representative recordings of two consecutive AP showing AP alternans (PCL = 250 ms) in control (black) and 0.3 μM E‐4031 (red) conditions. PCL: pacing cycle length. (d) Scatter plot of individually measured APD_80_ for reconstruction of restitution curve under the S1S1 stimulation. For each PCL, 20 raw data points (i.e., 10 odd beats and 10 even beats) were shown. (e) Mean value of APD_80_ (based on data shown in Figure 1d) for the reconstruction of APD restitution curve under the S1S1 stimulation. (f) Measured APD alternans amplitude (including averaged alternans and alternans maximum) relative to control for administration of 0.1 μM (*n* = 6, *p < 0.05), 0.3 μM (*n* = 5, *p < 0.05 and **p < 0.01), and 1 μM E‐4031(*n* = 5, *p < 0.05). (g) Measured changes in the PCL threshold for the onset of AP alternans relative to control for administration of 0.1 μM (*n* = 6, *p < 0.05), 0.3 μM (*n* = 5, *p < 0.05), and 1 μM E‐4031 (*n* = 5, **p < 0.01). (h) Representative APD restitution curves by S1S2 stimulation for control and administration of E‐4031. DI: diastolic interval (i.e., PCL‐APD_80_). (i) Maximum slopes of the APD restitution curves for control and administration of 0.1 μM (*n* = 5, **p < 0.01), 0.3 μM (*n* = 5, **p < 0.01), and 1 μM E‐4031(*n* = 5, **p < 0.01). (j) Measured ERP for control and administration of 0.1 μM (*n* = 6, **p < 0.01), 0.3 μM (*n* = 5, **p < 0.01), and 1 μM E‐4031 (*n* = 5, **p < 0.01). ERP: effective refractory period. For each cell, only a single drug concentration was used. Compared with control, *p < 0.05, **p < 0.01.

A whole‐cell patch clamp technique was used to record action potentials (AP_s_) of ventricular myocytes in current‐clamp mode with an Axopatch 700B amplifier and the Axon Digidata 1440A interface (Axon Instruments). AP recordings were lowpass filtered at 5 kHz and digitized at 10 kHz. Patch clamp pipettes (2–4 MΩ filled with internal solution) were pulled from borosilicate glass capillaries (WPI) with a horizontal puller P‐97 (Sutter Instruments). For all current‐clamp experiments, pipettes were filled with internal solution containing (mmol/L): 110 K‐aspartate, 20 KCl, 10 HEPES, 5 EGTA, 5 Na_2_‐phosphocreatine, and 5 MgATP, 0.1 NaGTP (pH adjusted to 7.2 with KOH). The chamber was perfused with heated Tyrode solution (37°C, PCTC2001, MappingLab Ltd.) mentioned above. After the re‐introduction of Ca^2+^ to a final concentration of 1.8 mM, the follow‐up electrophysiological experiments were carried out in ventricular myocytes with long rod, adherent cells, clear cardiac muscle cross striation, strong refractive, and no spontaneous contraction (As shown in Figure 1a).

### 2.5 Experimental protocol

AP_s_ were evoked by stimulation pulses with a duration of 4 ms and an amplitude of 1000 pA at 1 Hz. For S1S1 and S1S2 stimulus protocol, AP_s_ were evoked by stimulus pulses with a magnitude 1.5 times higher than the AP activation threshold at around 1500 pA. To evoke AP alternans, the stimulus frequency (for the S1S1 protocol) was increased by gradually reducing the stimulus time interval from 450 ms by a step of −10 ms to the one until a 2:1 response (stimuli versus evoked action potentials) onsets. At each of the stimulus frequency, 22 S1 stimuli were applied. The stimulus protocol was repeated after administration of E‐4031 (MCE). Three drug concentrations (0.1, 0.3, and 1 μM) were used in this study.

The standard S1S2 stimulation protocol consisted of a train of 15 regular pulses (S1) at a stimulus time interval of 500 or 1000 ms, followed by a premature extra‐stimulus (S2) with a progressively reduced coupling interval (−10 ms) from 350 ms to that one when the refractoriness was reached. The stimulus protocol was repeated after infusion of E‐4031. Similarly, three drug concentrations (0.1, 0.3, and 1 μM) were used. For each cell, only a single drug concentration and a programmed stimulus were used to maintain the data reliable. APD_80_ was measured as the duration from the overshoot to 80% percentages of repolarization analyzed by clampfit 10.7 (Molecular devices).

### 2.6 Optical mapping

#### 2.6.1 Optical mapping system

Two 530 nm LEDs (LEDC‐2001, MappingLab Ltd.) were used to illuminate the heart after the emissions were bandpass filtered (530 ± 20 nm) to minimize stray of excitation light reaching the dye. The fluorescence was passed through a long‐pass filter (550 nm) followed by a dichroic mirror with a cutoff (638 nm). For voltage signal recording, fluorescence with a wavelength above 638 nm was passed through a long‐pass filter (700 nm) and then imaged by the high‐speed camera (OMS‐PCIE‐2002, MappingLab Ltd.). For calcium signal recording, fluorescence with a wavelength below 638 nm was passed through a bandpass filter (585 ± 40 nm) and then imaged by high‐speed camera. The raw temporal resolution was 800 frames/second, and spatial resolution was 128‐by‐128 pixels, 2‐by‐2 cm^2^ field of view.

#### 2.6.2 Experimental protocol

When the isolated heart reaches a stable state, perfusing blebbistatin (10 μM, Abcam) from drug port was used to minimize contraction artifacts. RH237 (1 μg/mL, Santa) and Rhod2‐AM (1 μg/mL, Abcam) were perfused from drug port to enable simultaneous mapping of membrane potential and intracellular calcium transient. Before calcium dye loading, perfusion pluronic F127 (20% w/v in DMSO, Invitrogen) was used to aid calcium dye loading. After dye loading, the sinus node of the isolated heart was destroyed by electric soldering iron in order to eliminate the influence of its rhythm, such that a wide range of stimuli can be used. ECGs were recorded from two platinum electrodes placed on both sides of the heart (left ventricle & right atrium). Electrical stimuli (two platinum electrode) were delivered onto the apex of the heart by an isolated constant voltage/current stimulator (VCS3001, MappingLab Ltd.), and each of the stimulus pulses had a duration of 2 ms pulse and amplitude 1.5‐fold the diastolic stimulus threshold.

In the whole heart setting, to induce cardiac alternans by an incremental fast‐pacing protocol (S1S1), a train of 50 pulses per stimulus frequency was applied to the heart at the stimulation site, and the stimulation interval was progressively reduced from 450 ms (the longest pacing interval producing no ventricular escaped beats) down to about 120 ms till 2:1 response or even VF was induced under control conditions. The protocol was repeated after infusion of E‐4031 (0.1, 0.3, and 1 μM). Considering the effect of a duration of stimulation and attenuation of fluorescence signals, for each isolated heart only one drug concentration was used. For each pacing rate, a duration of 5–12 s video recordings of voltage and intracellular calcium signals was made. Once VF was induced, pacing protocol was terminated. With such experimental configuration, stable recordings of voltage (*V*_m_) and intracellular Ca^2+^ signals could be obtained for >2 h in preliminary experiments assuring the system's stability.

The analysis of raw data in optical mapping experiments was carried out by using Omapscope5.0 software (Mappinglab Ltd.). APD_80_ was measured as the duration from the maximum amplitude of the *V*_m_ to 80% percentages of repolarization. Activation maps were generated using (dF/dt)_max_. At least 5% of the mapping areas having in‐phase changes of the APD alternans (∆APD_80_ ≥ 3 ms), a feature defined as SCA. Two consecutive (odd/even) beats showed out‐of‐phase changes of the APD alternans (∆APD_80_ ≥ 3 ms) for at least 5% of the mapping areas, a feature defined as SDA. Alternans vulnerable windows (AVWs) was calculated as T1–T2, with T1 referring to the large PCL at which AP alternans begins, and T2 referring to the small PCL at which AP alternans ends. Alternans onsets (PCLonset) means the PCL at which AP alternans (∆APD_80_ ≥ 3 ms) occurs in continuous 6 beats. APs of the last 10 beats at a given stimulation PCL (i.e., 41th–50th S1 stimuli) were used for calculate APDsmall/CaDsmall and APDlarge/CaDlarge, and further to analysis spatiotemporal heterogeneity of APD/CaD alternans in different PCLs and region of the ventricle. APDsmall/CaDsmall was calculated as the average of the 5 smaller APDs/CaDs, and APDlarge/CaDlarge was calculated as the average of the 5 larger APD/CaD. The APD/CaD spatial dispersion over the whole mapping tissue was calculated by the range interquartile (IQR = Q3–Q1) of the box plot drawn by APD_80_ and CaD_80_ of the last 38 beats of a sequence of stimuli.

### 2.7 Statistical analysis

The original action potential data obtained by whole‐cell patch clamp experiment were analyzed by clampfit 10.7 (Molecular devices) for *n* (specified later) number of individual cells. The analysis of AP and Ca signals was carried out by using Omapscope5.0 software (Mappinglab Ltd.) built in the optical mapping experiment for *n* (specified later) number of isolated hearts. Non‐linear curve fitting for the APD and CV restitution curve was performed using OriginPro 8.0 (Origin Lab). Statistical comparisons were evaluated by using unpaired Student's *t*‐test and with repeated measures ANOVA, as appropriate, followed by the Newman–Keuls test. Group data were expressed as mean ± SEM. A value of *p* < 0.05 was considered significant. In the figures, the designations for *p* values are as follows: *p < 0.05, **p < 0.01, and ***p < 0.001, respectively.

## 3 RESULTS

### 3.1 AP alternans at the cellular level

Figure 1 presents the recorded membrane APs and the effect of E‐4031 on modulating their APDs from healthy guinea pig ventricular myocytes, which have characteristics of long rod, adherence, clear cardiac muscle cross striation, strong refractive, and no spontaneous contraction after re‐introducing Ca^2+^ to electrophysiological concentration (1.8 mM) as shown in Figure 1a. Figure 1b shows the representative Aps recorded at 1 Hz in control, 0.3 μM E‐4031 administration and washout conditions. E‐4031 caused a remarkable APD prolongation (the measured APD_80_ was increased by about 32%), which was reversible by washout. Such E‐4031 induced APD prolongation was concentration dependent. Administration of 0.1, 0.3, and 1 μM E‐4031 increased the APD_80_ (1 Hz) to be 1.24 ± 0.18, 1.37 ± 0.21 times and 2.02 ± 0.21 folds of the one in control, respectively. Cellular AP alternans were induced in both control and impaired repolarization conditions. Figure 1c shows the representative diagram of AP alternans before and after E‐4031 administration at pacing cycle length (PCL) = 250 ms. Two consecutive APs (evoked by odd and even stimulus) were shown, illustrating changes in their amplitudes and durations. It was shown that the administration of E‐4031 increased degree of the alternans as measured by the difference in APD_80_ of the two consecutive APs (∆APD_80_, control versus 0.3 μM E‐4031: 80 vs. 150 ms).

The occurrence of AP alternans was dependent on the pacing rate as shown in the APD restitution curves in Figure 1d,e for the measured individual and mean APD_80_ values. In this study, we defined the occurrence of AP alternans as ∆APD_80_ > 5 ms (∆APD_80_ = (Mean APD_80_)_odd_ − (Mean APD_80_)_even_; (Mean APD_80_)_odd_ and (Mean APD_80_)_even_ refer to the (Mean APD_80_ of 11 odd and 11 even stimuli in all 22 S1 stimuli), respectively). By gradually decreasing the PCL, a bifurcation point (i.e., the threshold of AP alternans genesis) in the APD restitution curve was observed. As shown in Figure 1d,e, administration of 0.3 μM E‐4031 shifted the bifurcation point to the right (i.e., at a larger PCL; PCL: 230 versus 260 ms for control versus 0.3 μM E‐4031), increased the alternans amplitude (averaged ∆APD_80_ in the alternans range: 48.73 versus 62.48 ms; alternans maximum, 86.47 versus 117.94 ms for control versus 0.3 μM E‐4031). Further statistical analysis results (Figure 1f) show that as compared to control condition, administration of E‐4031 (0.1, 0.3, and 1 μM) increased the alternans amplitude in the alternans average (by 1.34 ± 0.25 times (*n* = 6, *p < 0.05), 1.67 ± 0.46 times (*n* = 5, *p < 0.05), and 2.41 ± 1.05 times (*n* = 5, *p < 0.05), respectively); increased the alternan maximum (by 1.46 ± 0.44 times (*n* = 6, *p < 0.05), 1.75 ± 0.32 times (*n* = 5, **p < 0.01), and 2.86 ± 1.53 times (*n* = 5, *p < 0.05), respectively); and increased alternans PCL threshold (Figure 1g; by 17.50 ± 9.57 times (*n* = 6, *p < 0.05), 36.00 ± 19.49 times (*n* = 5, *p < 0.05), and 92.50 ± 28.723 times (*n* = 5, **p < 0.01), respectively), all of which were positively correlated with the degree of APD prolongation, suggesting AP alternans were more susceptible to be induced and the AP alternans amplitude was increased in impaired repolarization. All the measured differences were statistically significant compared with that before administration.

We further quantified the effect of APD prolongation on the maximum slope of the APD restitution curve, greater than one of which has been proposed as a potential measure to characterize the stability of the induced AP alternans. Results for control and impaired repolarization condition are shown in Figure 1h. It was shown that the APD restitution curve obtained by the standard stimulus (S1S2) protocol was steeper in administration of 0.1 μM E‐4031 (i.e., with a greater maximum slope; control versus 0.1 μM E‐4031: 2.19 vs. 4.36). Furthermore, the increase in the maximum slope of the APD restitution curve was positively correlated with the degree of APD prolongation as shown in Figure 1i (E‐4031 relative to control: 1.58 ± 0.31 times (*n* = 5, **p < 0.01), 2.25 ± 0.50 times (*n* = 5, **p < 0.01), and 2.75 ± 0.62 times (*n* = 5, **p < 0.01) for 0.1, 0.3, and 1 μM, respectively), and all the differences were statistically significant. The measured effective refractory period (ERP) was also positively correlated with the degree of APD prolongation as shown in Figure 1j (ΔERP, E‐4031 relative to control: 15.00 ± 5.48 ms (*n* = 6, **p < 0.01), 38.00 ± 8.37 ms (*n* = 5, **p < 0.01), and 80.00 ± 29.16 ms (*n* = 5, **p < 0.01) for 0.1, 0.3, and 1 μM, respectively).

### 3.2 AP alternans conduction at the tissue level

**FIGURE 2:**
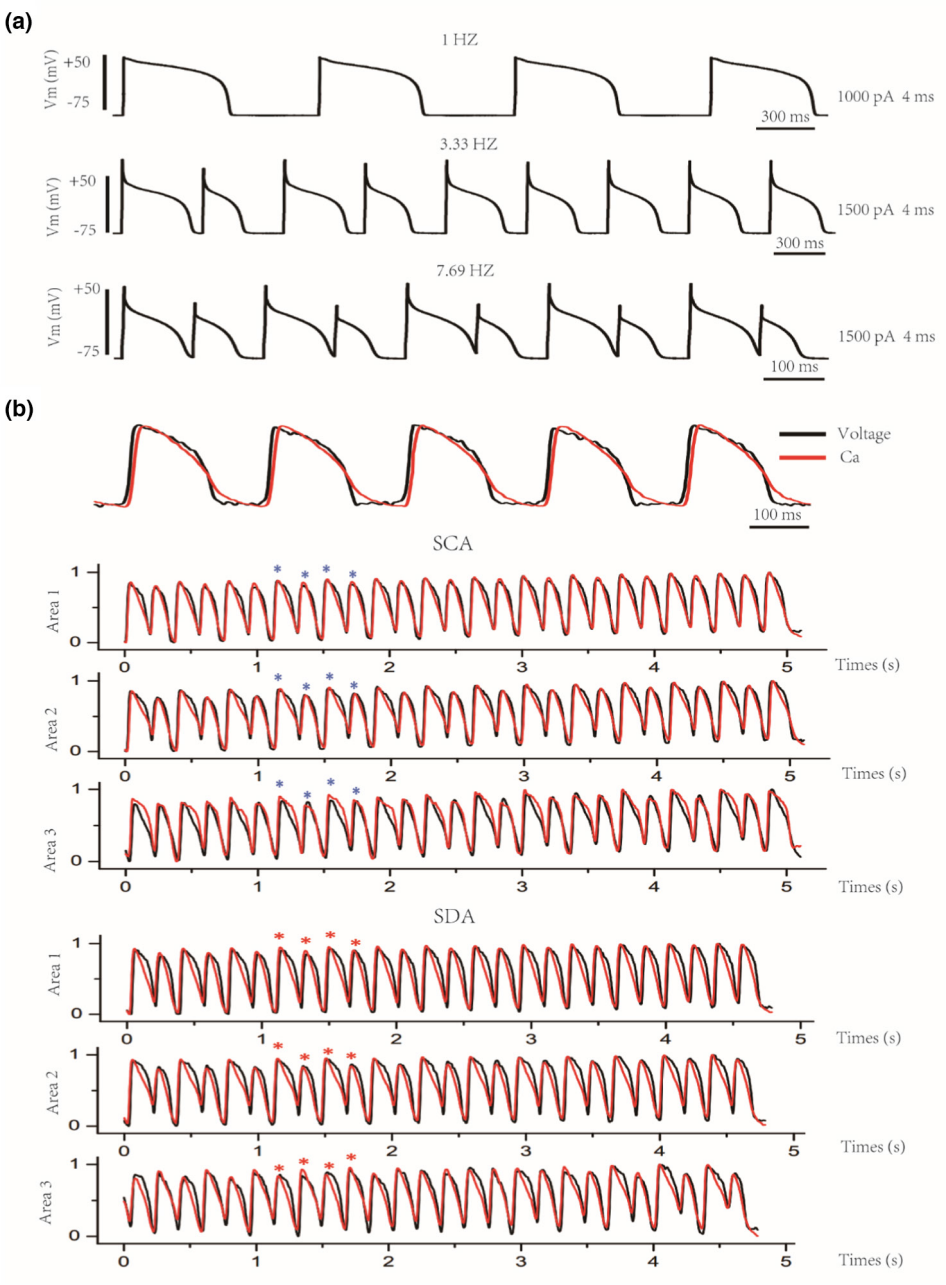
AP and Ca alternans at the cellular and tissue level measured by S1S1 stimulus in patch clamp and optical mapping technology. (a) AP alternans of ventricular myocytes in adult guinea pigs at higher stimulation frequencies. (b) The example of optical *V*_m_ and Ca signals of single pixel in optical mapping experiments (top). Cellular alternans be manifested as spatially concordant alternans (SCA, middle) and/or spatially discordant alternans (SDA, bottom) at the tissue level. As the blue star shows, AP and Ca alternans are in‐phase changes in whole tissue, that is, SCA; as the red star shows, AP and Ca alternans are out‐of‐phase changes in different regions, that is, SDA.

As shown in Figure 2 and our previous work (You et al., 2021), we measured AP and Ca alternans at the cellular and tissue level by S1S1 stimulus in patch clamp and optical mapping technology. TWA is measured as every‐other‐beat variations in action potential morphology (AP alternans; Figure 2a). By optical mapping experiments, Figure 2b (top) shows the diagram of optical Vm and Ca signals of single pixel, and at the tissue level, cellular AP alternans can be reflected as spatially concordant alternans (SCA), in which whole tissue exhibit AP alternans of in‐phase changes (Figure 2b (middle)) and/or spatially discordant alternans (SDA), in which APD in different regions exhibit APD alternans of out‐of‐phase changes (Figure 2b (bottom)). These observations were similar to a previous study in showing that cellular AP alternans can be manifested as SCA and/or SDA at the tissue level with an increase of pacing frequency (Colman, 2020).

**FIGURE 3:**
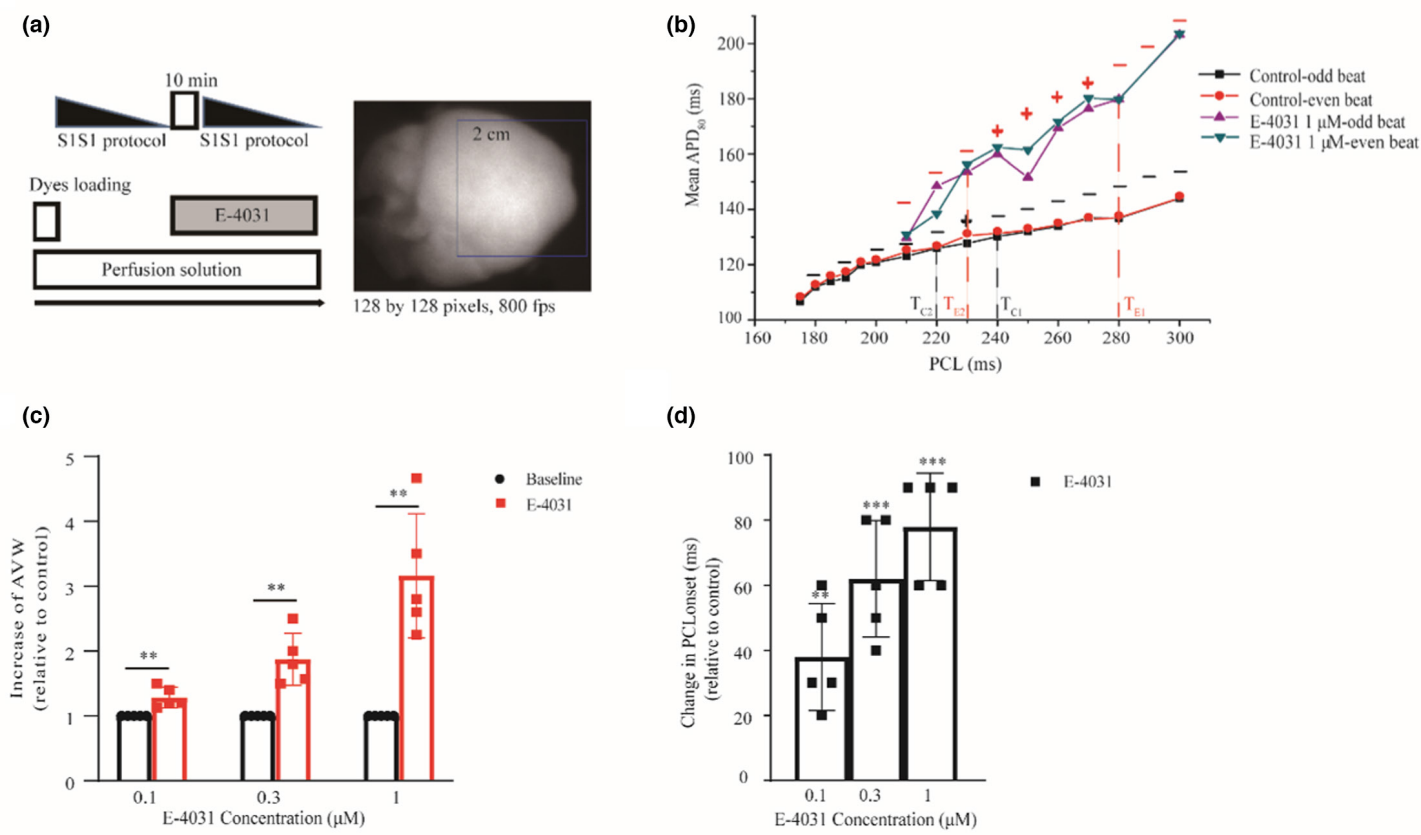
AP alternans induced at the isolated heart level for control and impaired repolarization conditions. (a) Experimental scheme of voltage RH237 and Rhod‐2am optical mapping protocol in isolated guinea pig hearts (left) and cardiac mapping area (right). (b) Representative plot of mean APD_80_ (averaged over 25 even for old beats from the whole mapping site) against PCLs in control and E‐4031 1 μM conditions. AP alternans occurred in a range of PCLs as marked (+, indicates AP alternans at that PCL; −, indicates no AP alternans at that PCL). (c) AVW for control and 0.1 μM (*n* = 5, **p < 0.01), 0.3 μM (*n* = 5, **p < 0.01), and 1 μM E‐4031 (*n* = 5, **p < 0.01) conditions. AVW was calculated as T1–T2, where T1 refers to the large PCL at which AP alternans begins, and T2 refers to the small PCL at which AP alternans ends. (d) Threshold of AP alternans as measured by the PCL at which alternans onsets (PCL_onset_) for control and administration of 0.1 μM (*n* = 5, **p < 0.01), 0.3 μM (*n* = 5, ***p < 0.001), and 1 μM E‐4031 (*n* = 5, ***p < 0.001). For each isolated heart, only a single drug concentration was implemented. Data were compared with control, *p < 0.05, **p < 0.01, ***p < 0.001.

To further investigate how the AP alternans at the cellular level were reflected in the tissue level, membrane voltage and intracellular calcium dual‐optical mapping were used, and results are shown in Figure 3 for control and impaired repolarization conditions. Figure 3a shows the schematic diagram of the optical mapping experimental protocols (left) and the mapping area (right) of the heart. At the isolated guinea pig heart levels, with normal KH solution, administration of E‐4031 also prolonged APD, causing an increase of APD_80_ (registered at whole mapping site; at 3 Hz) by 1.21 ± 0.04 times, 1.23 ± 0.06 times, and 1.43 ± 0.09 times of the control value for 0.1, 0.3, and 1 μM E‐4031, respectively. The role of such prolonged APD in regulating the threshold for generating AP alternans and alternans vulnerable windows (AVWs) at the tissue level was further investigated, and results are shown in Figure 3b–d.

Figure 3b plots the mean APD_80_ averaged across the whole tissue of consecutive 25 odd and 25 even beats under control and E‐4031 1 μM conditions against PCLs. Alternating APD_80_ between old and even beats occurred in a range of PCLs (+, indicated AP alternans at that PCL; −, indicated no AP alternans at that PCL; AP alternans criteria: the difference of mean APD_80_ > 3 ms). The result showed that 1 μM E‐4031 increased the APDs at each stimulus PCL with a greater APD prolongation at large PCLs, and more obvious APD_80_ difference between the odd and even beats as compared to the control condition. Figure 3c shows an increase in AVW by administration of E‐4031. AVW was calculated as T1–T2, with T1 referring to the large PCL at which AP alternans begins, and T2 referring to the small PCL at which AP alternans ends. As shown in the figure, AVW increased in impaired repolarization and was positively correlated with the degree of prolonged APD compared with control (E‐4031 relative to control, AVW was increased by 1.29 ± 0.16 times (*n* = 5, **p < 0.01), 1.87 ± 0.40 times (*n* = 5, **p < 0.01), and 3.16 ± 0.96 times (*n* = 5, **p < 0.01) for 0.1, 0.3, and 1 μM, respectively). Figure 3d also shows that prolonged APD promoted the occurrence of AP alternans, manifested by a shift of the bifurcation point to the right, suggesting AP alternans was induced at a larger PCL (E‐4031 relative to control, the PCL at which alternans onsets (PCL_onset_) was increased by 38.00 ± 16.43 ms (*n* = 5, **p < 0.01), 62.00 ± 17.89 ms (*n* = 5, ***p < 0.001), and 78.00 ± 16.43 ms (*n* = 5, ***p < 0.001) for 0.1, 0.3, and 1 μM, respectively).

### 3.3 Conduction alternans and conduction block

**FIGURE 4:**
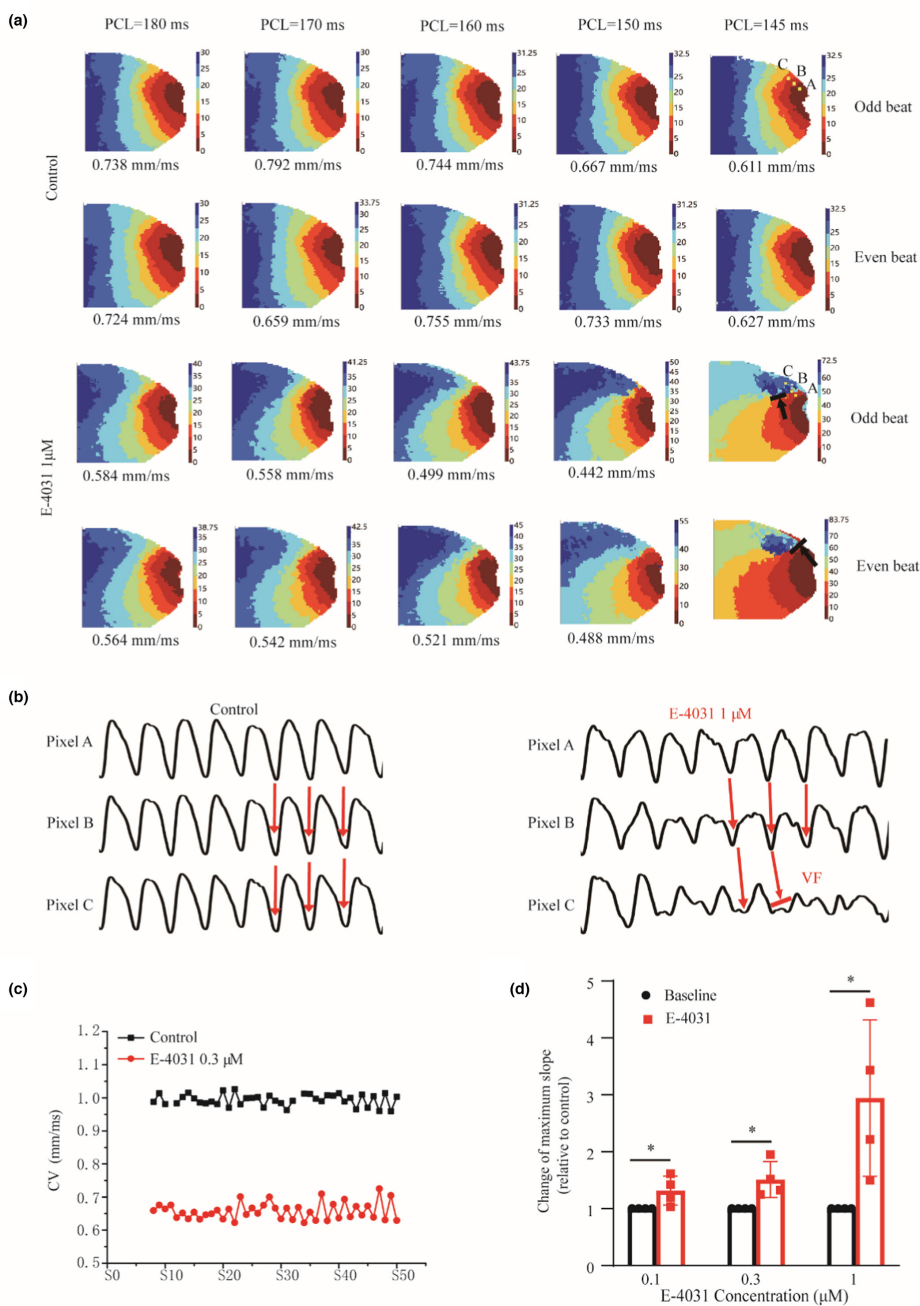
Voltage mapping of AP conduction pattern and the occurrence of conduction block in control and E‐4031 conditions at the tissue level. (a) Representative AP activation timing sequence for two consecutive beats (odd and even) in the tissue with PCL changing from 190 to 145 ms (for each PCL, data 7 s after the first stimulus were shown). Measured mean conduction velocity across the tissue was shown as numbers under the activation map. (B) Representative voltage time series recorded from of three pixels points (A, B, C; marked in yellow; as shown in Figure 3a) at PCL = 145 ms for 8 consecutive beats. Red arrows indicate the direction of conduction, VF: ventricular fibrillation. (c) Representative time course of measured CV (globally averaged) at PCL = 190 ms for 8th to 50th of consecutive S1 stimulation. (d) The maximum slope of the CV restitution curve for control and 0.1 μM (*n* = 4, *p < 0.05), 0.3 μM (*n* = 4, *p < 0.05), and 1 μM E‐4031 (*n* = 4, *p < 0.05) conditions. For each isolated heart, only a single drug concentration was used. Data were compared with control, *p < 0.05.

Figure 4 shows that AP alternans at the cellular level were reflected as conduction alternans at the tissue level, leading to possible regional conduction block that formed a substrate for reentry. Figure 4a shows the AP conduction pattern illustrated by color‐coded activation timing sequences, measured conduction velocity (CV; globally over the mapping tissue) and their rate‐dependence (i.e., CV for different PCLs from 180 to 145 ms) for odd and even beats in control and 1 μM E‐4031 conditions. In control, AP evoked by a localized stimulus (i.e., the dark red region) spread out as a target pattern, with an almost the same CV for the odd and even beats for large PCLs, but obvious alternating CVs between the two for small PCLs. The measured CV was rate‐dependent as it decreased with a decreased PCL. In the E‐4031 condition, the heterogeneous conduction became more pronounced, manifested as a more curved wavefront for all PCLs as compared to control. With a decreased PCL, such heterogeneous conduction became more pronounced, leading to localized conduction block at PCL < 150 ms.

Such localized conduction block was illustrated in Figure 4b, which shows representative time traces of APs recorded from three sites (pixels A, B, and C marked by the yellow dot in the vicinity of conduction block zone) at PCL = 145 ms. In control condition, though alternating, 1:1 conduction of APs at the three sites illustrated by a phase delay (red arrow) among them was observed with out‐of‐phase feature (i.e., SDA). In E‐4031 condition, such SDA became more pronounced, leading to a regional conduction block as shown by second red arrow in the AP time trace of site C. In this case, the corresponding excitation wavefront at the vicinity tissue region encircled site C and re‐enter the region after it became excitable after a time delay, leading to VF due to the formation of sustained reentrant excitation as manifested by the irregular AP time series from site C.

The mechanism of conduction alternans leading to possible conduction in impaired repolarization was further examined. Results are shown in Figure 4c for the representative CV time course measured (globally averaged) from the 8th to 50th S1 stimulation at the PCL = 190 ms. The result showed that the CV was obviously varying from beat to beat in both control and E‐4031 conditions, with a marked every‐other‐beat alternans for the latter. Slow CV was observed for a small AP, leading to conduction delays that gave the rest of the tissue more time to recover from previous excitations. When the smaller AP excitation waves reached the more recovered tissue part, the AP evoked became larger and conducts more rapidly. Thus, AP alternans manifests as CV alternans at the tissue level, leading to formation of SDA and the occurrence of conduction block at sufficiently high stimulation frequency in impaired repolarization.

We further explored the causal relationship between the formation of SDA as well as spontaneous arrhythmogenesis and the slop of the CV restitution curve. The result showed that the CV restitution curve was steeper in impaired repolarization (maximal slope for control versus 0.3 μM E‐4031: 0.012 vs. 0.016). Figure 4d shows that the maximum slope of CV restitution curve was greater in E‐4031, and the increase of the maximum slope of CV restitution curve was positively correlated with the degree of APD prolongation (E‐403 relative to control, maximal slope was 1.32 ± 0.26 times (*n* = 4, *p < 0.05), 1.51 ± 0.32 times (*n* = 4, *p < 0.05), and 2.94 ± 1.38 times (*n* = 4, *p < 0.05) of the control for 0.1, 0.3, and 1 μM, respectively). These results indicated that the steep CV restitution curve was the key determinant leading to SDA and spontaneous transition from AP alternans to VF in impaired repolarization.

### 3.4 Spatiotemporal heterogeneity of APD, CaD alternans in impaired repolarization

**FIGURE 5:**
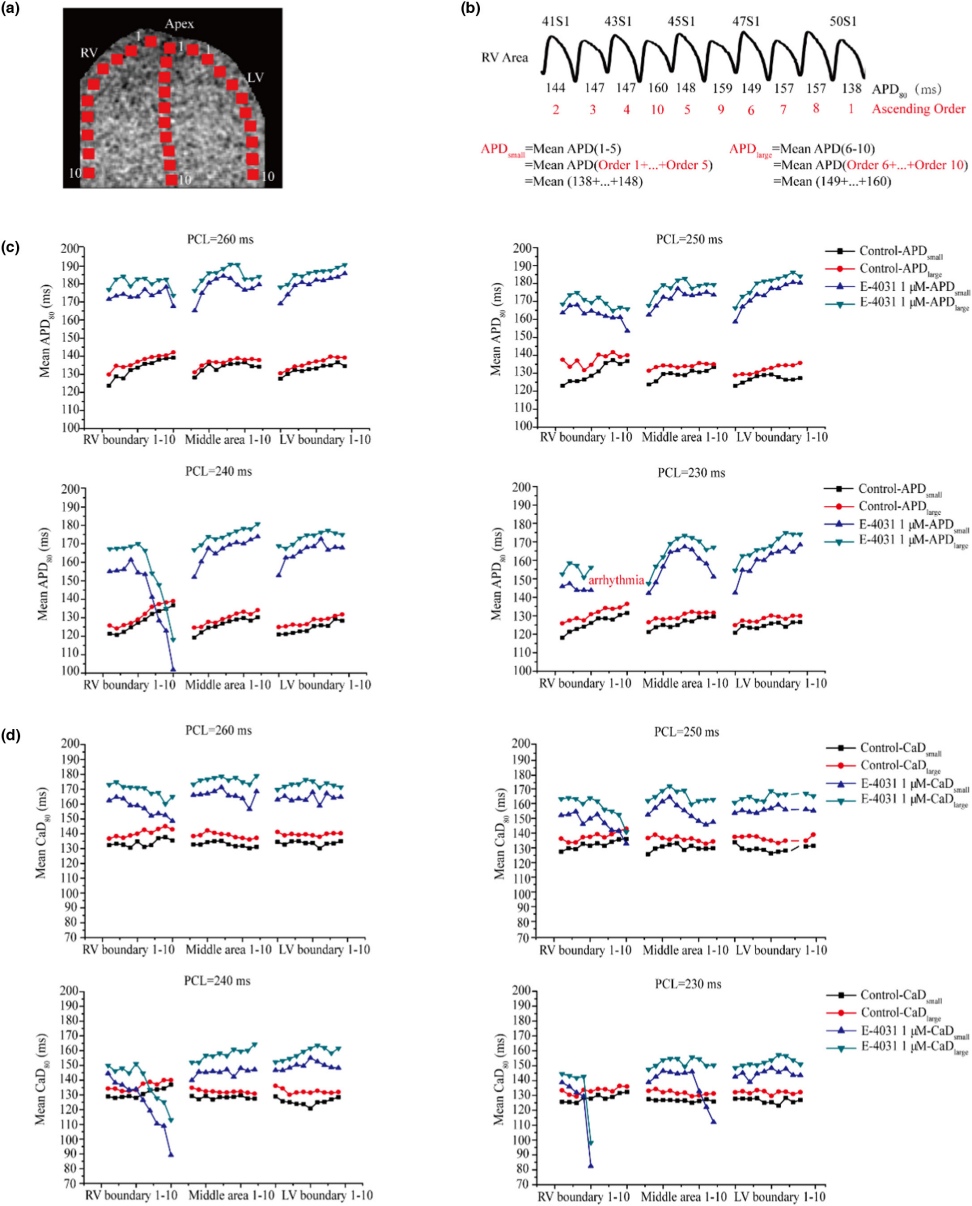
Spatiotemporal heterogeneity of APD and CaD over the mapping tissue in control and E‐4031 condition. Impaired repolarization augmented regional difference in APD and CaD. (a) Representative diagram of APD, CaD recording sites over the whole mapping tissue area (totally 30 regions) along three recording lines (marked by square symbols). RV: right ventricular, LV: left ventricular. (b) Time series of 10 recorded APs and explanation of method used to analyze the spatial heterogeneity of APD alternans in a recording site; the last 10 beats (i.e., 41th–50th S1 stimuli) at a given PCL were used to analyze to compute APD_80_, which were ordered by their values ascendingly from 1 (smallest APD) to 10 (largest value) as marked by red numbers below the APD values. APD_small_ was calculated as the average of the 5 smaller APDs, and APD_large_ was calculated as the average of the 5 larger APD. (c) Time series of APDs for 10 of each of the three recording lines (a total of 30 recording sites) under PCL = 260, 250, 240, and 230 ms. PCL: pacing cycle length. (d) the corresponding time series of CaD for the 30 recording sites under PCL = 260, 250, 240, and 230 ms.

We further analyzed the spatiotemporal heterogeneity in the APD and CaD distributions over the tissue when SDA was formed. Figure 5a shows schematically the 10 recording sites along each of the three different conduction pathways, one along the left ventricular boundary, one in the middle of the heart and the third along right ventricular boundary. For each recording site, APs of the last 10 beats at a given stimulation PCL (i.e., 41th–50th S1 stimuli) were used for analysis (Figure 5b). For each of the APs, APD_80_ was computed, and the 10 APDs were ranked by their values ascendingly from 1 to 10 (red label below APD values). APD_small_ was computed as the average of the 5 small APDs (i.e., APD_small_ = Mean APD (1–5)), and APD_large_ was computed as the average of the 5 large APDs (APD_large_ = Mean APD (6–10)). The spatiotemporal heterogeneity of the CaD distribution was obtained using the same method as above: the CaDs of the last 10 beats in each recording site at a given stimulation PCL were obtained and ordered, and then, we calculated CaD_small_ = Mean CaD (1–5) and CaD_large_ = Mean CaD (6–10; data not shown). Figure 5c shows the time course of the spatial distribution of computed APD_small_ and APD_large_ for the 30 recording sites at different PCLs (PCL = 260, 250, 240, and 230 ms). The result showed marked difference between APD_small_ and APD_large_, which varied from beat to beat (temporal variation), and varied among recording sites alone the same recording line and between different recording lines (spatial variations). Such spatiotemporal heterogeneity was augmented by decreasing the PCL. The smaller the PCL, the marked spatiotemporal heterogeneity was observed. It was also augmented by the administration of 1 μM E‐4031 as at the same PCL more pronounced spatiotemporal APD variations were observed. Interestingly, at the region near the basal of the RV boundary, more pronounced spatiotemporal APD heterogeneity was observed in impaired repolarization. With a fast‐pacing rate (i.e., PCL at 240 ms), a marked reduction in the APD near the basal region of the RV boundary in impaired repolarization was observed with time, leading to conduction failure at PCL = 230 ms, which led to spontaneous formation of VF.

Same as the spatiotemporal heterogeneity of APD alternans, Figure 5d shows the accompanied spatiotemporal heterogeneity of CaD alternans in total 30 recording sites for variant PCLs (PCL = 260, 250, 240, and 230 ms). The results showed that the spatiotemporal heterogeneity of CaD was also augmented in impaired repolarization. At PCL = 230 ms, a significant increase in spatiotemporal heterogeneity near the basal region of the RV boundary (PCL = 240 ms) was observed before the onsetting of ventricular arrhythmias. Furthermore, the result showed that the spatiotemporal heterogeneity of CaD was more marked than that of APD, indicating that the instability of calcium handling dynamics may play an important role in arrhythmogenesis (Figure 5c,d).

### 3.5 Augmented spatial dispersion of APD and CaD in impaired repolarization

**FIGURE 6:**
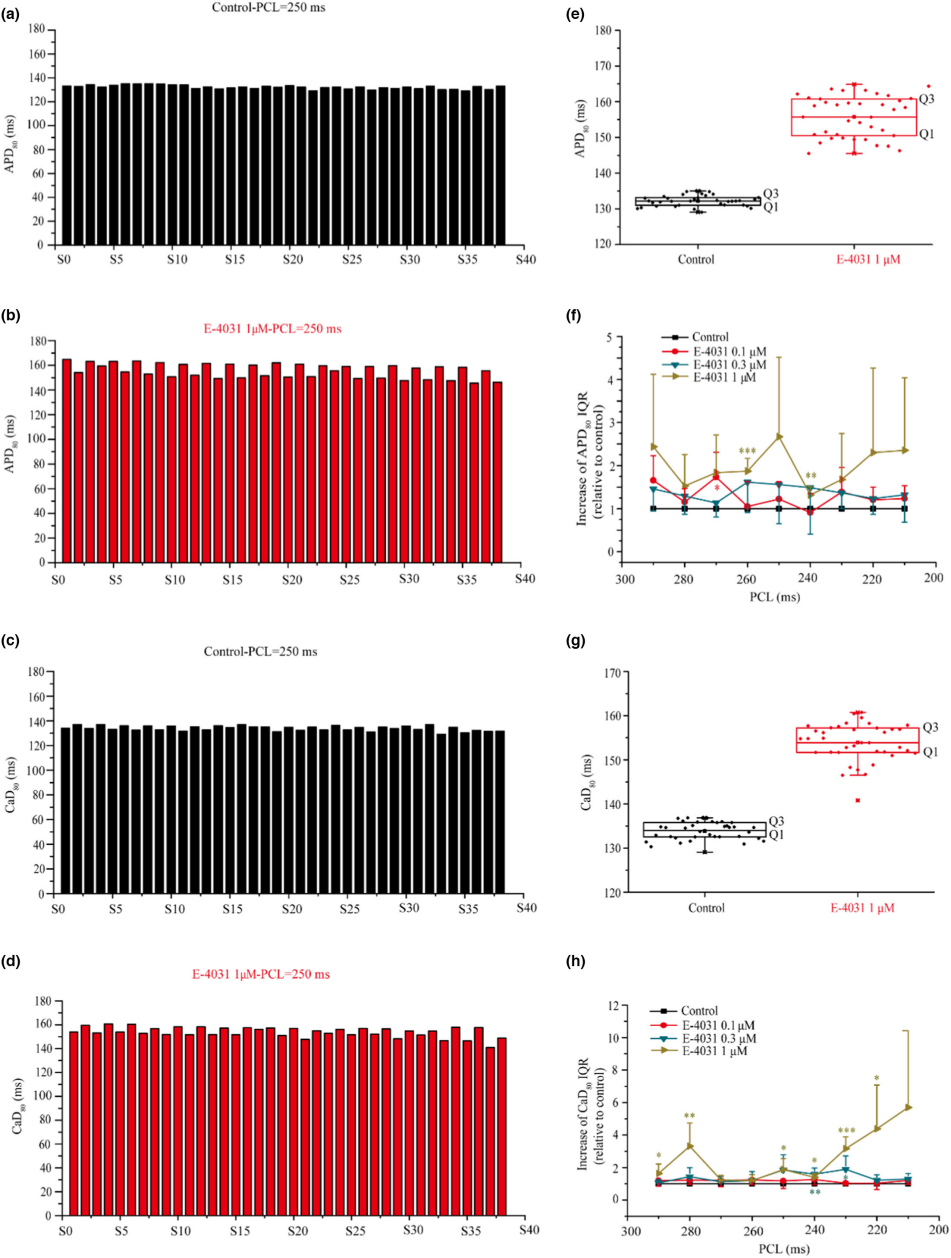
Impaired repolarization augmented the spatial dispersion of APD and CaD in isolated hearts. (a) Representative APD_80_ time series for the last 38 consecutive beats in control condition at PCL = 250 ms for analyzing APD spatial dispersion. (b) Representative APD_80_ time series of the last 38 consecutive beats after administration 1 μM E‐4031 at PCL = 250 ms. (c) The corresponding CaD_80_ of the last 38 consecutive beats in control (at PCL = 250 ms). (d) The corresponding CaD_80_ of the last 38 consecutive beats after administration 1 μM E‐4031 at PCL = 250 ms. (e) The box plot for the APD spatial dispersion (measured by range interquartile of the box plot: IQR = Q3–Q1) before and after administration E‐4031 (at PCL = 250 ms). The black and red dot means measured APD_80_ of the last 38 beats of a sequence of stimuli before and after administration 1 μM E‐4031. (f) Relative rate‐dependent increase of APD spatial dispersion in 0.1 μM (*n* = 4), 0.3 μM (*n* = 5), and 1 μM (*n* = 5) E‐4031 compared to that in control were for PCL changing from 290 ms to 210 ms. Compared to control, **p < 0.01, ***p < 0.001. (g) The box plot for the CaD spatial dispersion before and after administration E‐4031 (at PCL = 250 ms). The black and red dot means measured CaD_80_ of the last 38 beats of a sequence of stimuli before and after administration 1 μM E‐4031. (h) Relative rate‐dependent increase of CaD spatial dispersion in 0.1 μM (*n* = 4), 0.3 μM (*n* = 5), and 1 μM (*n* = 5) E‐4031 compared to that in control were for PCL changing from 290 ms to 210 ms. Compared to control, *p < 0.05, **p < 0.01, ***p < 0.001. For each isolated heart, only a single drug concentration was used.

Whether the formation of SDA augments the spatiotemporal heterogeneity of AP/Ca in impaired repolarization, we further analyzed the measured APD_80_ and CaD_80_ of the last 38 beats of a sequence of stimuli before and after administration at PCL = 250 ms. Figure 6a–d shows representative time courses of APD_80_ (Figure 6a,b) and CaD_80_ (Figure 6c,d) from a same recording site in control (Figure 6a,c) and 1 μM E‐4031 (Figure 6b,d) conditions, where prolonged APD and CaD, increased beat‐to‐beat APD/CaD variations in amplitude were observed in E‐4031. The APD/CaD spatial dispersion over the whole mapping tissue at PCL = 250 ms were shown by box plot (Figure 6e,g), indicating an increase of them (range interquartile: IQR = Q3‐Q1) after 1 μM E‐4031 administration (APD spatial dispersion: 2.14 ms vs. 10.29 ms for control vs. 1 μM E‐4031; CaD spatial dispersion: 3.25 ms versus 5.52 ms or control vs. 1 μM E‐4031). The APD and CaD spatial dispersion at different stimulation PCLs were further analyzed. As shown in Figure 6f,h, in the range of PCL = 290–210 ms, the APD and CaD spatial dispersion were significantly increased after administration E‐4031 and is positively correlated with the degree of APD prolongation (at PCL = 250 ms, APD spatial dispersion in relative to control is 1.22 ± 0.41 times (*n* = 4), 1.56 ± 0.91 times (*n* = 5), and 2.67 ± 1.85 times (*n* = 5) for 0.1, 0.3, and 1 μM E‐4031, respectively). With the increase of stimulation frequency (i.e., a decrease in PCL), the increasing trend of APD and CaD spatial dispersion was more obvious after administration E‐4031, implicating an increased arrhythmia susceptibility.

### 3.6 Increased susceptibility of ventricular arrhythmias in impaired repolarization

**FIGURE 7:**
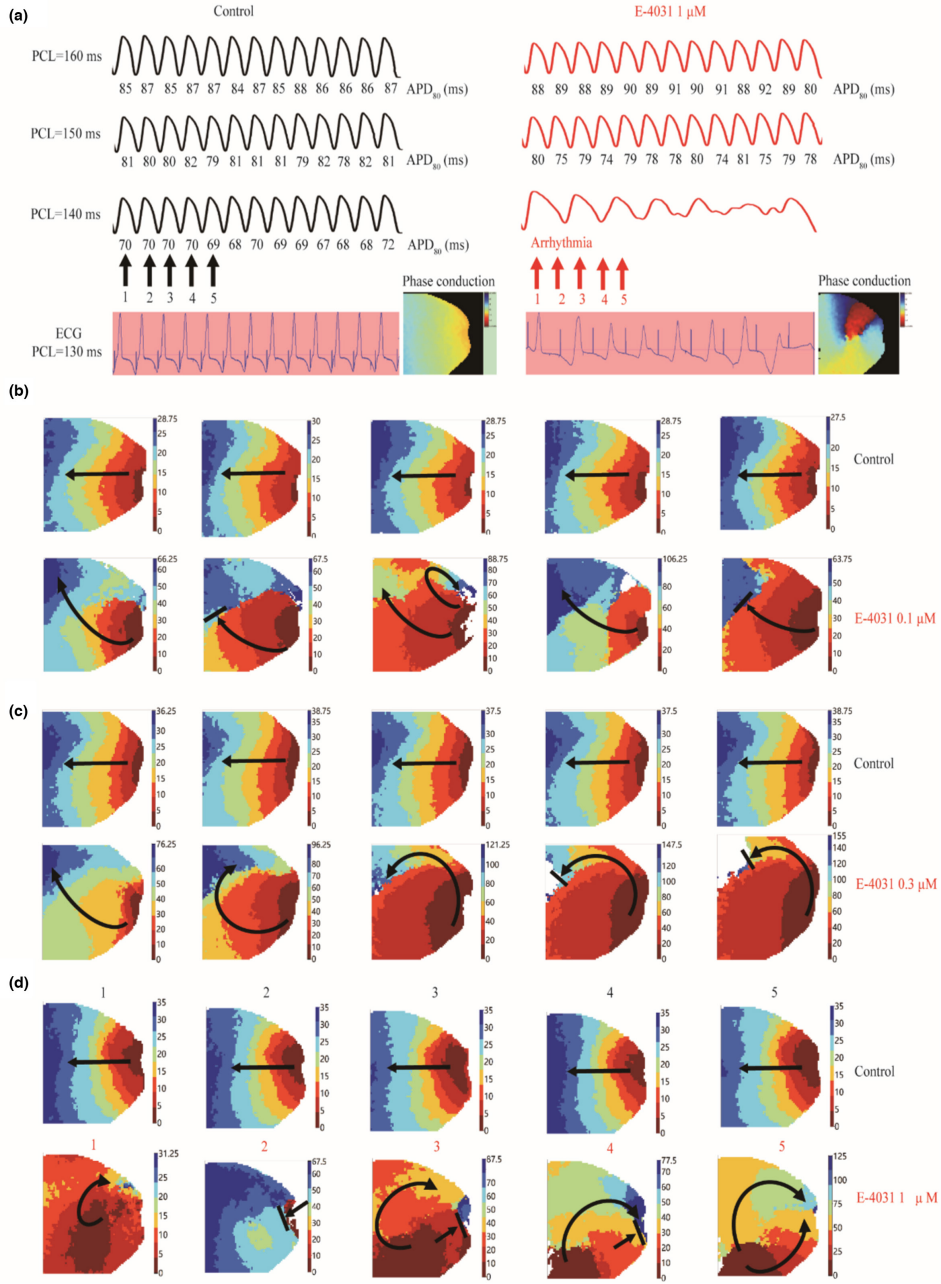
Increased susceptibility to ventricular arrhythmias in impaired repolarization. (a) Recorded AP time traces for 13 consecutive beats at PCL = 160, 150, and 130 ms for control and E‐4031 concentrations. The electrocardiogram at PCL = 130 ms and phase conduction before and after 1 μM E‐4031 administration was also shown. Values under the AP waveform were measured APD_80_. (b) Representation of AP activation pattern for 5 consecutive beats in control and 0.1 μM E‐4031 at PCL = 140 ms. (c) Representation of AP activation pattern for 5 consecutive beats in control and 0.3 μM E‐4031 at PCL = 170 ms. (d) The corresponding activation conduction patterns for 5 consecutive beats (1–5) as marked by the arrows in Figure 6a for control and 1 μM E‐4031 at PCL = 130 ms. (Phase conduction videos for control and 0.1, 0.3, and 1 μM E‐4031 are presented in Video S1). Five isolated hearts were used for each of the 0.1, 0.3, and 1 μM E‐4031 and each isolated heart only a single drug concentration was used.

Finally, optical mapping was used to assess the susceptibility to arrhythmia in impaired repolarization. Results are shown in Figure 7. Figure 7a shows representative recordings of 13‐beats APs recorded from whole mapping tissue in control (left panels) and 1 μM E‐4031 (right panels) conditions at PCL = 160 ms, 150 ms, and 140 ms. In the figure, the value below the AP time traces was measured APD_80_. With the increase of stimulation frequency (i.e., a decrease in PCL), AP alternans became more marked as shown by the difference in two consecutive APDs. In the control condition, it occurred at PCL = 232.0 ± 32.7 ms in control (5 isolated hearts were used for each of the 0.1, 0.3, and 1 μM E‐4031), but no ventricular arrhythmia was observed in any isolated hearts. With administration of 1 μM E‐4031, AP alternans occurred at PCL = 310.0 ± 28.3 ms (*n* = 5), representing an increased PCL_onset_ (i.e., increased inducibility) as compared to control. At PCL = 132.5 ± 16.0 ms, VF was observed in 4 of 5 isolated hearts arising from conduction block (see the ECG and conduction phase map before and after administration of 1 μM E‐4031. For conduction phase video, see Video S1).

Figure 7b shows the representation AP activation conduction pattern for the 5 consecutive beats in control and 0.1 μM E‐4031 conditions. In the figure, arrows illustrated the direction of excitation wave conduction. With 0.1 μM E‐4031 administration (Figure 7b), the conduction of 5 consecutive beats in control was stable, along the conduction direction from apex to base; however, the conduction was markedly disrupted that caused conduction block. In this case, in 1 of 5 isolated hearts, conduction block was induced leading to ventricular arrhythmia at PCL = 140 ms, but no VF was observed in the control condition (for phase conduction video see Video S1).

Figure 7c shows the representation AP activation conduction pattern of the 5 consecutive beats in control and 0.3 μM E‐4031 conditions (for phase conduction video see Video S1). With 0.3 μM E‐4031 administration, in 2 of 5 isolated hearts conduction block and ventricular arrhythmias were observed at PCL = 170.0 ± 0.0 ms, but no VF was observed in the control condition.

Figure 7d shows the corresponding AP activation conduction patterns for consecutive 5 beats as marked by 1–5 (black for control and red for E‐4031) as shown in the third row of Figure 7a for control and 1 μM E‐4031 conditions. With 1 μM E‐4031 administration (Figure 7d), the conduction was markedly disrupted that caused conduction block, leading to formation of excitation reentry at PCL = 130 ms. Collectively, these results suggested that the prolonged APD in impaired repolarization was associated with increases an increased susceptibility to ventricular arrhythmia.

## 4 DISCUSSION

### 4.1 Major findings

TWA has been used as a noninvasive biomarker for predicting the risk of fatal cardiac arrhythmias in many cardiac disease conditions, especially in LQTS (Grabowski et al., 2004; Huang et al., 2016). In this presented experimental model of impaired repolarization, we found that APD was prolonged at the cellular and tissue levels, which was accompanied by an increased tissue's vulnerability for the genesis of AP alternans and the initiation/maintenance of reentrant excitation waves (i.e., an increased risk of arrhythmogenesis) as compared to the control condition. Our major findings are as follows: (1) under the impaired repolarization, AP alternans at the cellular level and their conduction alternans (CV alternans) at the tissue level were more inducible and pronounced, leading to increased susceptibility of ventricular arrhythmias arising from a spontaneous transition from cellular AP alternans to reentrant excitation without need of premature stimulus; (2) such spontaneous transition from cellular AP to VF is attributable to a combined action of rate‐dependent APD and CV restitution properties (APDr and CVr), as well as the spatial–temporal heterogeneity of tissue's electrical activities; (3) the spatial–temporal heterogeneity of AP/Ca alternans augmented the spatial dispersion of excitation, leading to regional conduction block, forming a substrate conducive for arrhythmogenesis. These results provide first hand experimental evidence for demonstrating a spontaneous transition from cellular AP alternans to VF without the involvement of extra and premature trigger, and mechanistic insights into understandings of such transition.

### 4.2 Mechanistic link between TWA and arrhythmogenesis in impaired repolarization

TWA has been used as a biomarker indicating cardiac electrical instability that predisposes to malignant arrhythmia and even SCD in a variety of cardiac diseases (Fukaya et al., 2019; Holley & Cooper, 2009; Huang et al., 2018; Yang et al., 2016), including the LQTS (Grabowski et al., 2004; Terentyev et al., 2014). In some cases, such as increased beat‐to‐beat variability of ventricular repolarization duration, this could also be beat‐to‐beat variability of repolarization, which has been shown to be driven by alternative mechanisms than alternans (Johnson et al., 2010). Hormones have specific roles in modulating cardiac electrophysiological parameters and arrhythmia vulnerability, and estrogen likely exacerbates the breakdown of normal cardiac electrical activity in the presence of QT‐prolonging drugs (Yang et al., 2010). Though a large number of studies have attempted to elucidate the mechanisms underlying the genesis of TWA (i.e., AP alternans) at the cellular level (Qu et al., 2000; Song et al., 2018; Tse et al., 2016), however, it still remains unclear about possible mechanism(s) responsible for the transition of AP alternans at the cellular level to arrhythmias at the tissue level as premature stimulus was applied in those studies to initiate reentrant excitation. At the cellular level, the genesis of AP alternans can be attributable to dynamical restitution properties of cardiac tissue (such as the steep APD restitution; Grabowski et al., 2004; Nolasco & Dahlen, 1968), instable intracellular calcium cycling (Goldhaber et al., 2005; Song et al., 2018; Terentyev et al., 2014), and possible autonomic regulation (Nemec et al., 2003; Schwartz & Malliani, 1975). At the tissue level, the conduction of the AP alternans is influenced by the tissue's CV restitution property (Huang et al., 2020; Mironov et al., 2008; Wei et al., 2015), leading to the formation of SCA/SDA that amplifies the pre‐existing tissue heterogeneity (Tse et al., 2016) and substrates favoring the initiation and maintenance of arrhythmias. Data presented in this study provided experimental evidence in showing that the increased susceptibility of AP alternans is associated with a steeper APD restitution curve (>1) in impaired repolarization as compared to the control condition, due to a prolonged APD that slowed down the repolarization process. In the case of a prolonged APD, the time interval between the next excitation and the completion of the current AP repolarization is reduced, leading to a less time for ion channels to recover from activation. Consequentially, the AP evoked by the next excitation is small with a shorter APD due to the refractory property of cardiac tissue, leaving a longer DI before the following next excitation that allows more time for ion channels to recover to generate a large AP for the following up excitation. As such, alternating small and large APs are generated in response to a series of excitation pulses. In the case when the maximum slope of the APD restitution curve is less than 1, the generated alternans is transient and short lasted. However, when the maximum slope of the APD restitution curve is greater than or equal to 1, AP alternans with stable or even complex patterns can be generated (Colman, 2020; Huang et al., 2016). In the present study, our data showed that the impaired repolarization prolonged APD, leading to a steeper APD restitution curve that promoted the occurrence of AP alternans. It not only increased the amplitude of AP alternans (measured by the ΔAPD), but also shifted the PCL threshold (i.e., the bifurcation point of the APD restitution curve) to the right, suggesting for generating AP alternans to a greater value (i.e., the genesis of AP alternans at more slower heart rates as compared to control; Figure 3).

Augmented electrical heterogeneity in cardiac tissue due to conduction of SDA alternans has been believed to be responsible for the increased arrhythmogenesis of cellular AP/Ca alternans. However, there is lack of experimental evidence to demonstrate a spontaneous transition from SDA to arrhythmia without a trigger of premature stimuli, though a recent simulation study (Wang et al., 2018) has shown it is possible. In this study, we observed such a spontaneous transition from cellular AP alternans to the formation of reentrant excitation at the tissue level, and analyzed the factors underlying such transition. Our experimental data showed that the cellular AP alternans were reflected as conduction alternans at the tissue level, leading to an augmented spatial–temporal heterogeneity of APD and CaD, which was more remarkable in impaired repolarization due to a steeper CV restitution curve. In the whole heart setting experiments, we have observed that AP alternans were evoked by a series of rapid pacing (Figure 2b). At the vicinity of the stimulation site, the evoked large APs propagated fast, but the small ones propagated slowly (arising from the CV restitution property), leaving the rest of the tissue more time to recover from a previous excitation. When the smaller AP excitation wave reaches the more recovered tissue, the evoked AP becomes larger and conducts rapidly until it reaches the refractory tail of the previous excitation, where a small AP was generated. Consequentially, an excitation wave with conduction alternans was generated leading to the formation of SDA featured by alternating regions of fast conduction large AP and slow conduction small AP. In impaired repolarization, the CV conduction alternans and the formed SDA were more remarkable (Figure 4c), which were attributable to the increased maximum slope of the CV restitution curve (Figure 4d).

We have shown that the stiff CV restitution property of tissue plays a key role in the formation of SDA formation, leading to complex spatiotemporal dynamics of cardiac excitation. This observation is consistent with previous studies (Hayashi et al., 2007; Mironov et al., 2008). Once formed, the SDA generated functional spatial heterogeneity in tissue's electrical activity, augmenting the pre‐existing tissue heterogeneity. With time, the spatial heterogeneity evolved into complex spatiotemporal heterogeneity. In the setting of impaired repolarization, the observed spatiotemporal heterogeneity of the APD/CaD alternans were more pronounced and regionally different (Figure 5). At the basal region of the RV ventricle wall, a marked spatiotemporal heterogeneity was observed. With time evolving, the localized spatial heterogeneity in cardiac excitation became critical and led to conduction failure. Excitation waves encircled this region and reentered it when the tissue's excitability was resumed. As such, reentry was formed and eventually evolved into VF (Figure 5) spontaneously. Furthermore, our results showed that the spatiotemporal heterogeneity of CaD was more remarkable than that of APD, indicating that the instable calcium handling is involved in the occurrence of arrhythmogenesis.

Spatial APD dispersion has been shown to play an important role in facilitating the spontaneous transition from premature ventricular complex (PVC) to arrhythmia in a previous study (Huang et al., 2016; Odening et al., 2013), in which early afterdepolarizations (EADs) was involved as a trigger for reentry initiation in the transgenic LQT2 rabbit heart model, and in other cases (with *I*_Ks_/*I*_Kr_ blockade or late‐*I*_Na_ augmentation, under the influence of beta‐adrenergic receptor stimulation), beat‐to‐beat variability of repolarization was observed, which was associated with other alternative mechanisms rather than alternans (Johnson et al., 2010). Our results presented here showed that even without the involvement of premature ventricular excitation (i.e., EADs), an augmented spatial APD dispersion arising from AP conduction alternans in impaired repolarization can also increase tissue's arrhythmia susceptibility, leading to spontaneous transition from cellular AP alternans to VF (Figure 7 and see Video S1). This study provides experimental data in elucidating possible mechanism(s) responsible for the transition from TWA (e.g., ventricular APD alternans) to VF without involvement of ectopic focal activity.

### 4.3 Relevance to previous studies

Previous studies have shown that TWA is a forewarning of instable ventricular excitation and malignant arrhythmias in patients with various clinical conditions, such as LQTS (Huang et al., 2016; Liu et al., 2018; Terentyev et al., 2014) and drug‐induced LQTS (Grabowski et al., 2004; Wegener et al., 2008), acute myocardial infarction (Puletti et al., 1980), and heart failure (Gold et al., 2008; Klingenheben et al., 2000). Using computational (Liu et al., 2012, 2018; Wang et al., 2018) and animal experimental animal models (Grabowski et al., 2004; Liu et al., 2018; Nemec et al., 2010; Terentyev et al., 2014), mechanisms underlying the transition from TWA (e.g., AP alternans) to VF genesis and maintenances in impaired repolarization settings have been partly investigated (Bayer et al., 2016; Hayashi et al., 2007). In an experimental model of transgenic LQT2 rabbit hearts, it was shown that the impaired cardiac repolarization produced dynamical instabilities of cardiac excitation, increasing the spatial dispersion of repolarization or pre‐existing tissue heterogeneity that promoted the spontaneous initiation of arrhythmias in the case when premature ventricular complex was produced (Huang et al., 2016). Similarly, EADs arising from aberrant RyR‐mediated Ca^2+^ releases (Liu et al., 2012; Terentyev et al., 2014) also helped the spontaneous initiation of arrhythmias in transgenic rabbit model of LQT2 syndrome. In addition, abrupt increases in sympathetic discharges as an extrastimulus have also been shown to perpetuate VF genesis and maintenance in impaired repolarization settings (Nemec et al., 2003; Schwartz & Malliani, 1975).

In the present study, we have shown that in the condition of impaired repolarization mimicked by administration of E‐4031, impaired *I*_Kr_ in the adult guinea pig ventricular cells and isolated hearts prolonged APD, resulting in AP alternans both at the cellular and tissue level. This observation is consistent with previous clinical and experiment studies of TWA in impaired repolarization settings (Grabowski et al., 2004; Liu et al., 2018). It also produced conduction alternans, leading to the formation of SDA, increasing the spatial dispersion of AP repolarization and heterogeneity of Ca transients. Such spatiotemporal heterogeneity was regional dependence, leading to a local conduction block that allows for the formation of reentrant excitation, even without the involvement of PVC or EADs. Though our previous study has shown such a spontaneous transition from AP alternans to reentry in a computer model of cardiac tissue (Wang et al., 2018), this study provides first hand experimental data in demonstrating such a transition. Therefore, it adds mechanistic insights to understanding the spontaneous transition from AP alternans to VF initiation, particularly in impaired repolarization settings.

### 4.4 Limitations

This study is limited to healthy guinea pig ventricular myocytes and isolated hearts. The impaired repolarization model was obtained by using E‐4031, which may be different to pathological hearts. Nonetheless, the model provides a general case to investigate the genesis of AP alternans and its transition to VF in the condition of impaired cardiac repolarization as consequence of I_Kr_ blocking. Whether or not our results are suitable for AP alternans genesis and transition to VF in the case of other impaired ionic currents remains to be further investigated. Another potential limitation of the present study is the existing optical mapping technology, which requires electromechanical dissociation to eliminate motion artifacts, which may affect calcium homeostasis. Nevertheless, this is currently the only technique that is available for simultaneously quantifying calcium dynamics with membrane voltage.

## 5 CONCLUSIONS

The findings of this study both substantiate the causal link between the TWA and APD elongation and provide mechanistic explanation for the spontaneous transition from AP alternans to arrhythmia in the form of impaired repolarization without the involvement of premature stimulus of EAD/DAD/PVC. It has shown that the spontaneous transition from AP alternans to VF is attributable to (i) a combined action of the stiff rate‐dependent APD/CV restitution properties of cardiac tissue; and (ii) the augmented spatial–temporal heterogeneity of AP/Ca alternans and their spatial dispersion, leading to either conduction failure or regional conduction block, forming a substrate conducive for arrhythmogenesis.

## AUTHOR CONTRIBUTIONS

Henggui Zhang conceived and designed the overall study. Tingting You performed the experiments and designed the experiments. Tingting You and Cunjin Luo wrote the manuscript. Kevin Zhang and Henggui Zhang edited and revised the manuscript. All authors analyzed and interpreted the data, and agreed to be accountable for all aspects of the work in ensuring that questions related to the accuracy or integrity of any part of the work are appropriately investigated and resolved. All persons designated as authors qualify for authorship, and all those who qualify for authorship are listed.

## FUNDING INFORMATION

This work was supported by the National Natural Science Foundation of China (No. 61803318) and Scientific‐Technological Collaboration Project under Grant (No. 2018LZXNYD‐FP02).

## CONFLICT OF INTEREST STATEMENT

The authors declare no conflict of interest.

## ETHICS STATEMENT

All procedures were performed following the protocol approved by the Animal Care and Use Committee of the Southwest Medical University with the grant number of swmu20210401.

## INSTITUTIONAL REVIEW BOARD STATEMENT

Not applicable.

## INFORMED CONSENT STATEMENT

Not applicable.

## Supporting information


Video S1–S6
